# Supplementary material for: LNK suppresses interferon signaling in melanoma
Source: Nat Commun. 2019 May 20;10:2230. doi: 10.1038/s41467-019-09711-y (PMC6527565; doi:10.1038/s41467-019-09711-y)
Supplement: Supplementary file 1 — Supplementary Information [file 41467_2019_9711_MOESM1_ESM.pdf]

Supplementary Information

LNK suppresses interferon signaling in melanoma

Ding, et al.

Supplementary Tables 1-3

Supplementary Figures 1-12

Supplementary Table 1. PCR primers used in the study.

Genes	Primer sequences	
Human LNK/DLW13	CACTTTCCCTCGGTCGTG	Real time PCR
Human LNK/DLW14	GGGACAGCCAGAAGAACTAA	Real time PCR
IFI6-RT-FP	CCATCTATCAGCAGGCTCCG	Real time PCR
IFI6-RT-RP	TTTCTTACCTGCCTCCACCC	Real time PCR
IFI27-RT-FP	CGGTGAGGTCAGCTTCACAT	Real time PCR
IFI27-RT-RP	TGGCCACAACTCCTCCAATC	Real time PCR
IFI35-RT-FP	GCAGAGCCTCCTGAGGTGTA	Real time PCR
IFI35-RT-RP	GCACTGAAAATGGGACCTTGT	Real time PCR
IFITM3-RT-FP	ATCCCAGTAACCCGACCGC	Real time PCR
IFITM3-RT-RP	CTGTCCCTAGACTTCACGGAG	Real time PCR
IDO1-RT-FP	TGGCCAGCTTCGAGAAAGAG	Real time PCR
IDO1-RT-RP	TGGCAAGACCTTACGGACATC	Real time PCR
ISG20-RT-FP	GACCTGAAGCACGACTTCCA	Real time PCR
ISG20-RT-RP	CAGGCTGTTCTGGATGCTCT	Real time PCR
CD274-RT-FP	TTTGCTGAACGCCCCATACA	Real time PCR
CD274-RT-RP	TTGGTGGTGGTGGTCTTACC	Real time PCR
mouse LNK CRISPR/ DLW1503	CACCGGCAGCTCTTCGATCCGCCCA	CRISPR for mouse LNK
mouse LNK CRISPR/ DLW1504	AAACTGGGCGGATCGAAGAGCTGCC	CRISPR for mouse LNK
DLW1175	CACCGTGAGTTGCACGCCGTAGCGG	CRISPR for human LNK sgLNK-1
DLW1176	AAACCCGCTACGGCGTGCAACTCAC	CRISPR for human LNK sgLNK-1
DLW1179	CACCGTGGAGCATGTCCACGACCGA	CRISPR for human LNK sgLNK-2
DLW1180	AAACTCGGTCGTGGACATGCTCCAC	CRISPR for human LNK sgLNK-2

Supplementary Table 2 sgRNA sequence targeting LNK.

CRISPR guide RNA	Sequences
Human sgLNK-1	TGAGTTGCACGCCGTAGCGG
Human sgLNK-2	TGGAGCATGTCCACGACCGA
Mouse sgLnk	GCAGCTCTTCGATCCGCCCA

Supplementary Table 3. Antibodies used in this study.

Antibodies		Identifier	Source
anti-human LNK	Polyclonal Sheep IgG	AF5888	R&D system Inc
anti-mouse/human LNK	mouse monoclonal IgG2b	(A-12)sc-393709	Santa Cruz Biotechnology, Inc.
anti-β-actin	Mouse Monoclonal IgG1	a1978	Sigma-Aldrich
anti-p-JAK2 (Tyr1007/1008)	Rabbit mAb	3776S	Cell signaling
anti-JAK2	Rabbit mAb	3230S	Cell signaling
anti-p-JAK1 (Tyr1022/1023)	Rabbit Polyclonal	3331	Cell signaling
anti-JAK1	Rabbit mAb	3344	Cell signaling
anti-p-STAT1 (Tyr701)	Rabbit mAb	7649	Cell signaling
anti-STAT1	Rabbit Polyclonal	9172	Cell signaling
anti-IRF-1	Rabbit mAb	8478	Cell signaling
anti-Cleaved Caspase-3 (Asp175)	Rabbit Polyclonal	9661	Cell signaling
anti-Cleaved Caspase-9 (Asp330)	Rabbit Polyclonal	9501	Cell signaling
anti-BCL-2	Rabbit mAb	4223	Cell signaling
anti-BIM	Rabbit mAb	2933	Cell signaling
anti-Cleaved PARP (Asp214)	Rabbit mAb	5625	Cell signaling
anti-PD-L1	Rabbit mAb	13684	Cell signaling
anti-TAP1	Rabbit Polyclonal	12341	Cell signaling
anti-GAPDH	Rabbit mAb	2118	Cell signaling
anti-PTPN2	Rabbit Polyclonal IgG	11214-1-AP	Proteintech
anti-Mouse Pd-1	Rat IgG2a, κ monoclonal	BE0146	Bio X cell
anti-Mouse Pd-l1	Rat IgG2b, κ monoclonal	BE0101	Bio X cell

Supplementary Figure 1

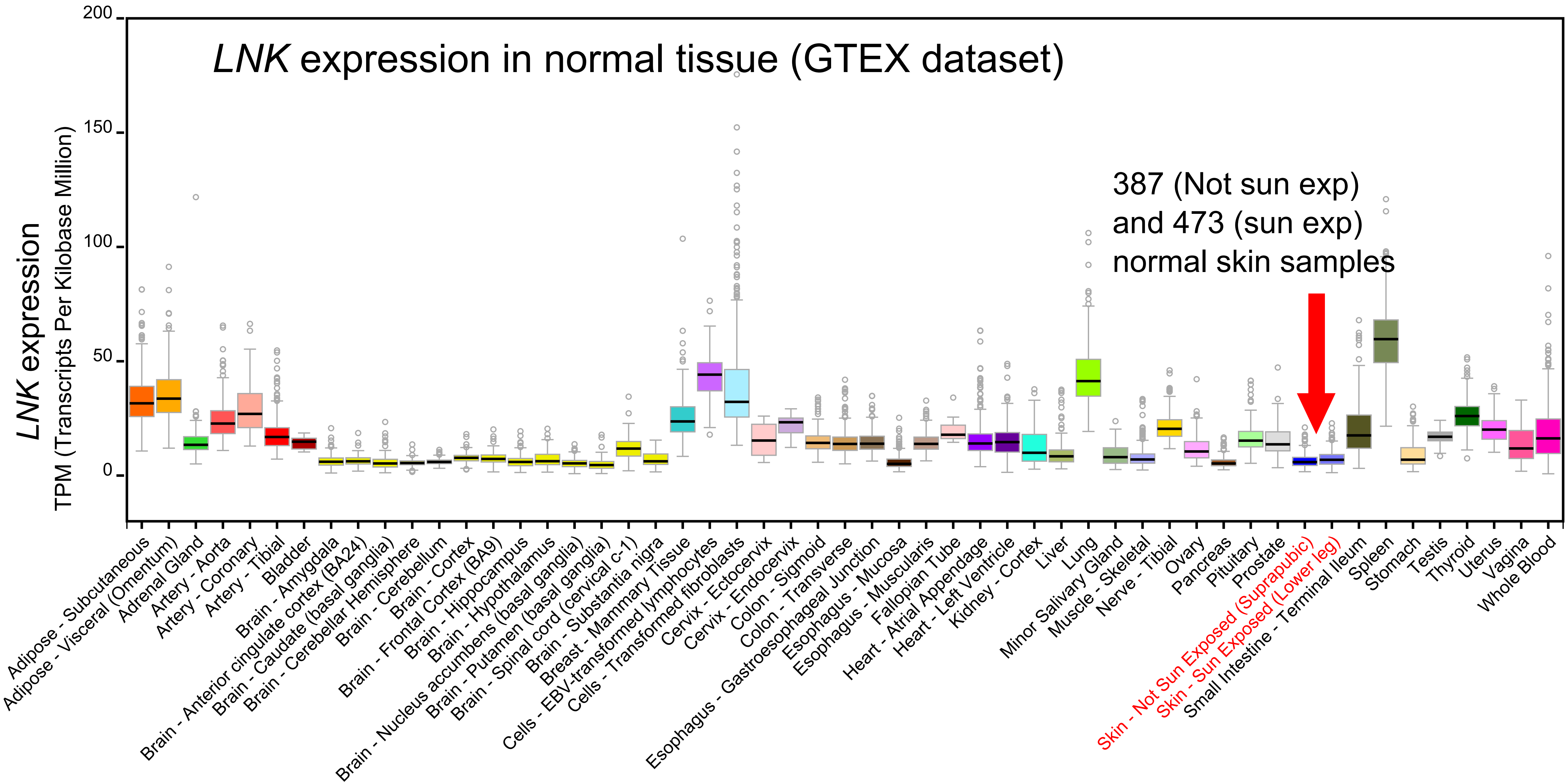

Supplementary Figure 1 *LNK* mRNA expression in normal skin. RNA sequencing data retrieved from GTEX database [https://www.gtexportal.org]. FPKM: Fragments Per Kilobase of transcript per Million mapped reads.

Supplementary Figure 2

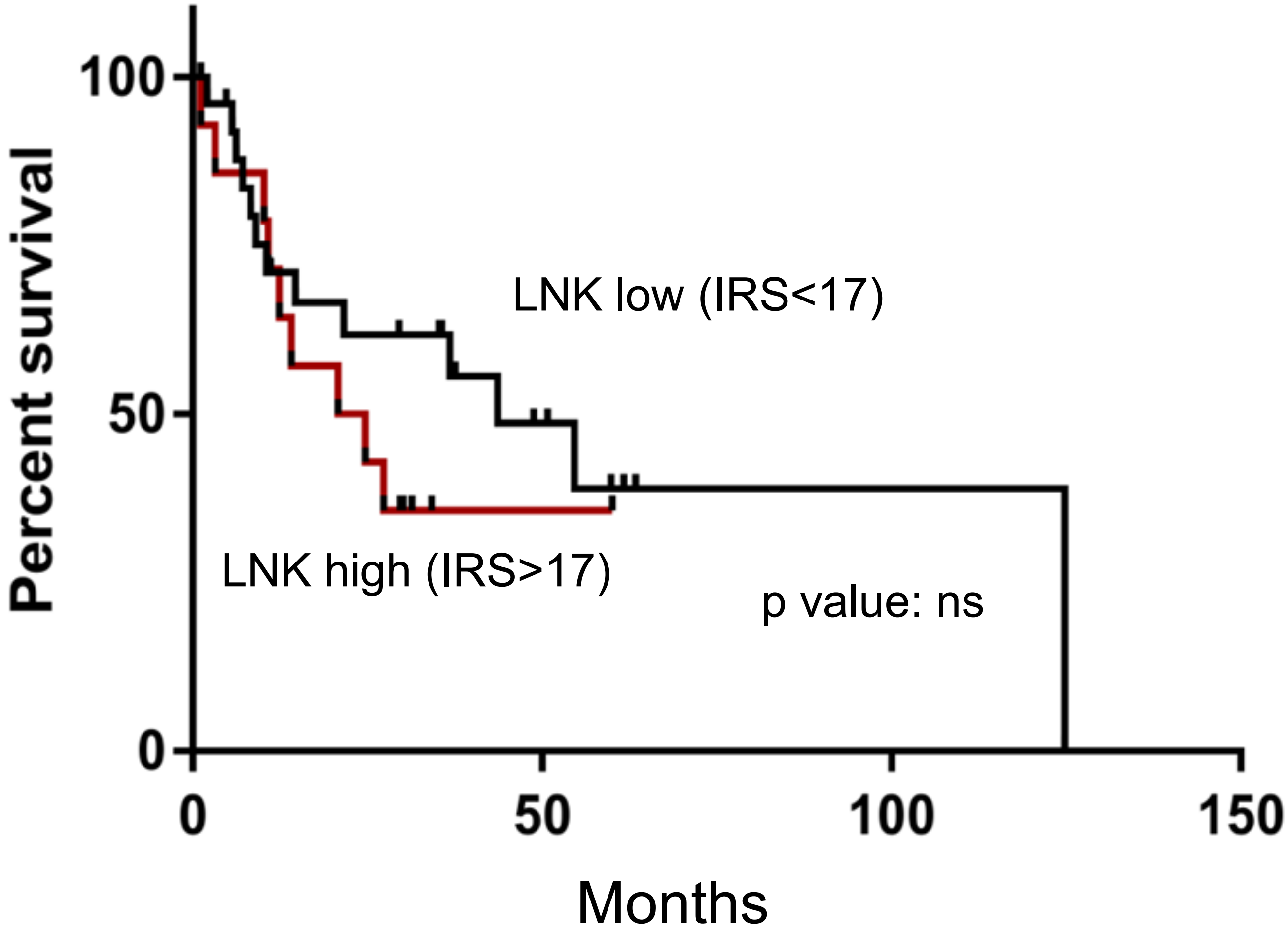

Supplementary Figure 2 Melanoma patients with high LNK expression in their tumors have inferior overall survival. LNK protein expression was determined by IHC staining in stage 3 regional lymph nodes metastasis melanoma samples (this stage contained largest number of patients for analysis). Patients were divided into high LNK (with an immunoreactive score (IRS) above 17, red line) and low LNK (IRS below 17, black line), and expression levels of LNK were correlated with overall survival (Kaplan-Meier curve).

Supplementary Figure 3

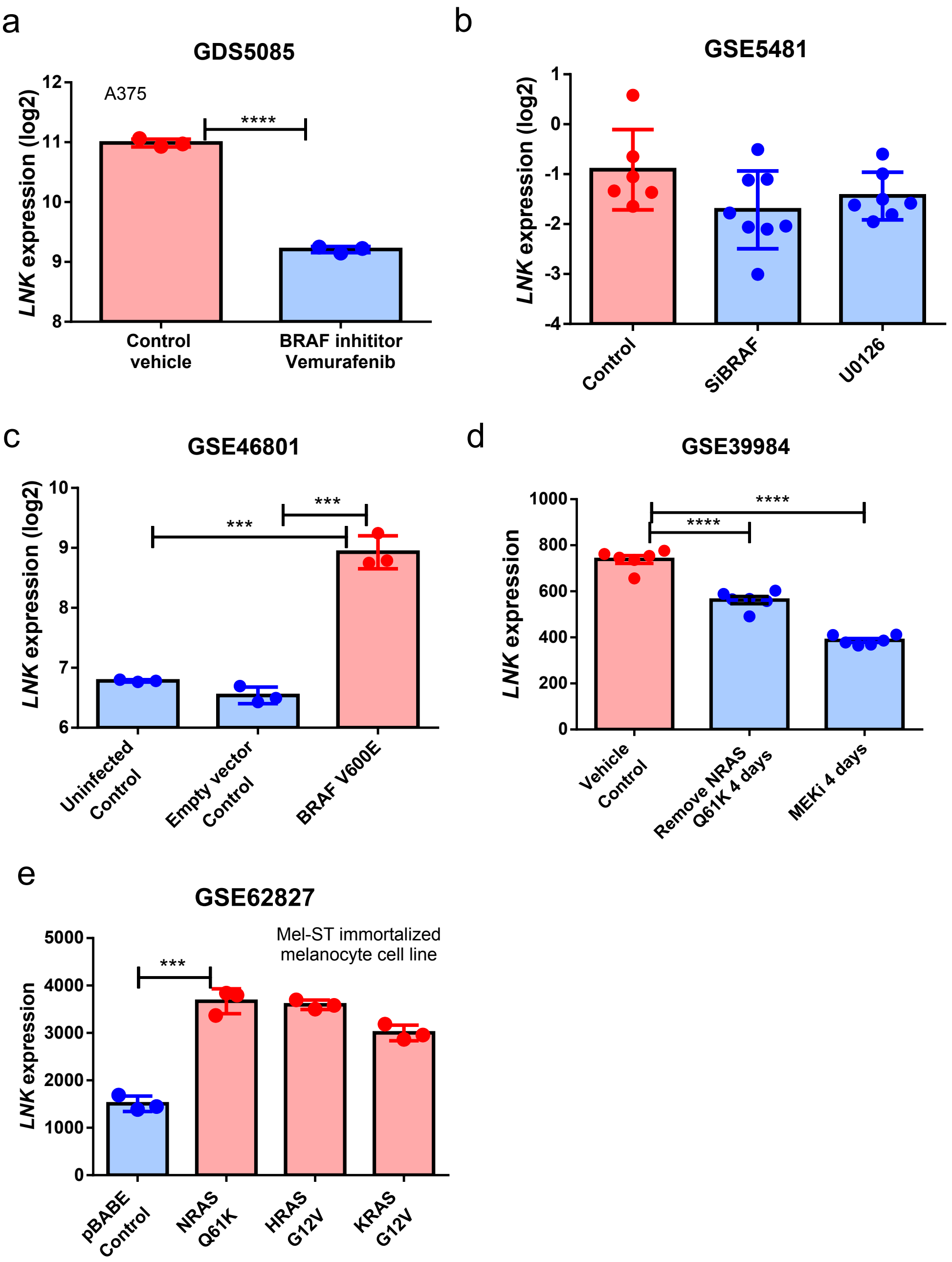

Supplementary Figure 3 *LNK* mRNA levels are associated with hyperactivated RAS-RAF-MEK signaling. **a** Inhibition of BRAF activity using Vemurafenib (PLX4032) down-regulated levels of *LNK* mRNA. Mean ± SD, n=3. ****, p<0.0001. **b** Inhibition of BRAF-MEK signaling using either *BRAF* siRNA or MEK inhibitor U0162 downregulated expression of *LNK* mRNA. Mean ± SD. **c** Force expression of a mutant BRAF (V600E) increased *LNK* levels. Mean ± SD. ***, p<0.001. **d** Inhibition of NRAS-MEK signaling by either withdrawing expression of a tetracycline inducible NRAS Q61K or using a MEK inhibitor (MEKi), downregulated *LNK* expression. Mean ± SD. ****, p<0.0001. **e** Forced expression of mutant RAS genes (either NRAS Q61K, HRAS G12V or KRAS G12V) in immortalized melanocyte cell line Mel-ST increased LNK expression. Mean ± SD. ***, p<0.001. All p values were calculated using unpaired two tailed t-test.

Supplementary Figure 4

a

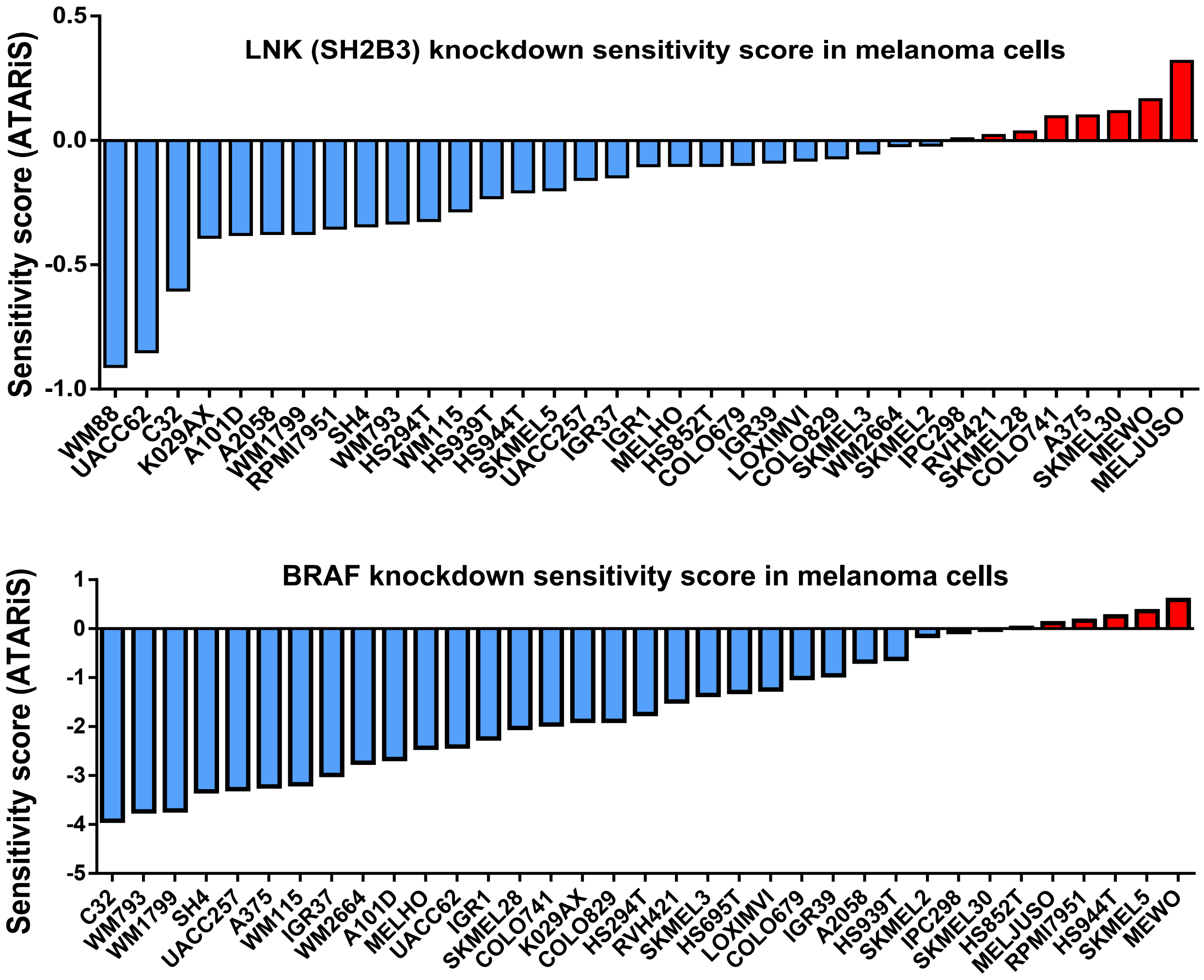

b

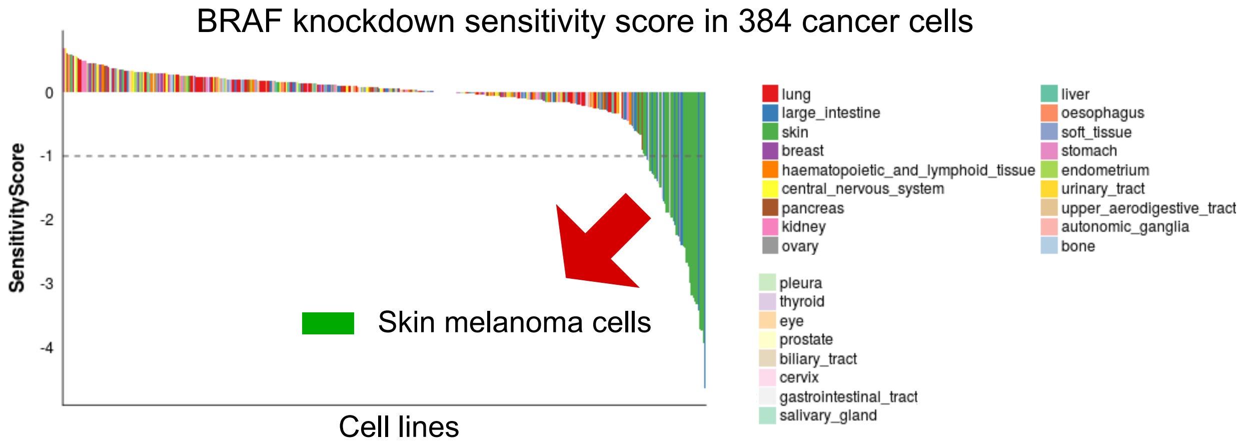

Supplementary Figure 4 ATARiS profiles of *LNK* and *BRAF* shRNA in Novartis cancer cell lines shRNA library project database (https://oncologynibr.shinyapps.io/drive/). **a** shRNA (~20 different shRNA targeting different region of each gene) targeting either *LNK* (upper panel) or *BRAF* (lower panel) were decreased/depleted in most of the melanoma cell lines (blue color) after 14 days post transduction. **b** ATARiS profiles of *BRAF* shRNA in 384 different cancer cell lines in Novartis cancer cell lines shRNA library project database.

Supplementary Figure 5

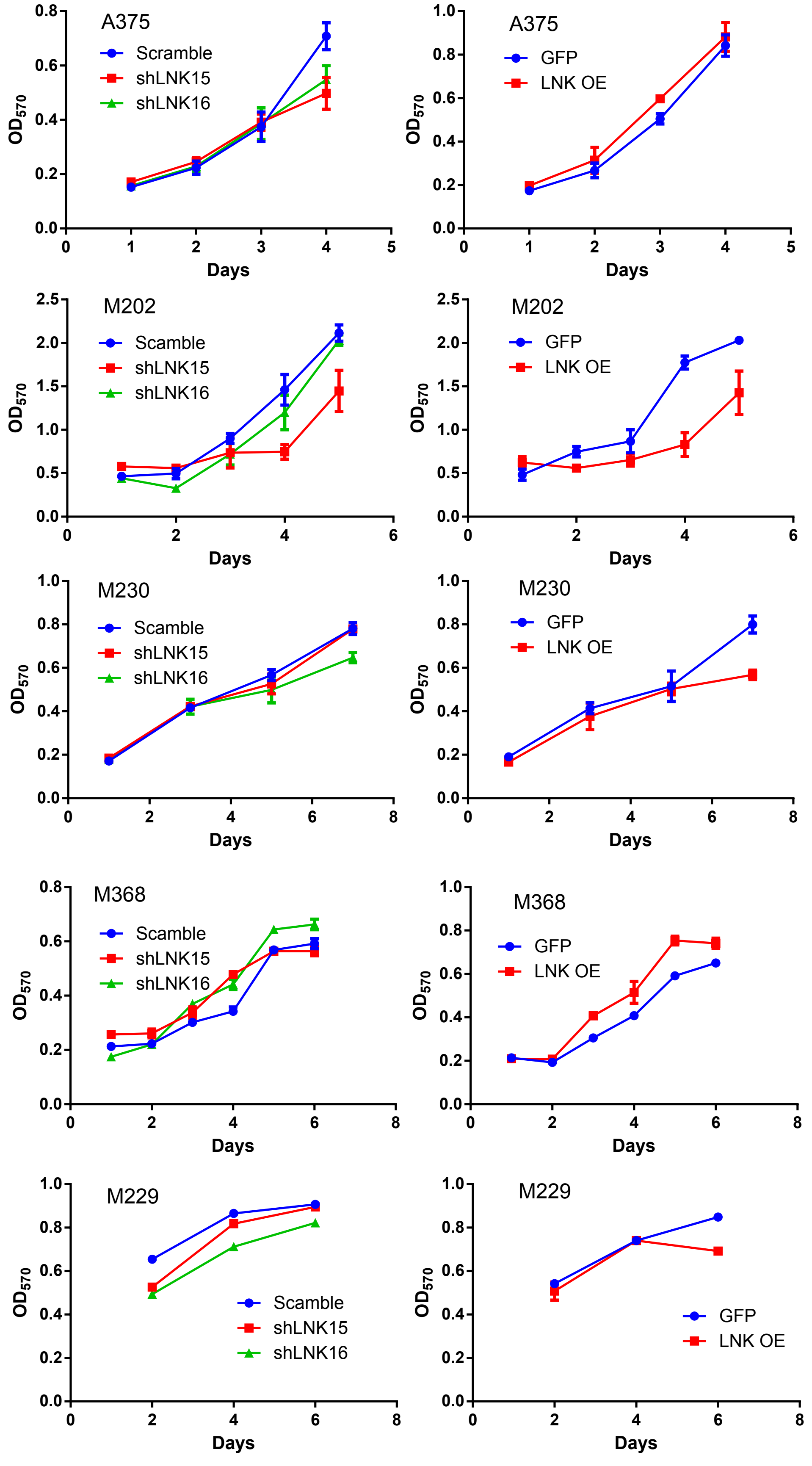

Supplementary Figure 5 MTT assays of melanoma cells after either silencing or overexpressing LNK. Scramble, non-target scramble shRNA control cells. GFP, control cells with forced expression of GFP. LNK OE, cells with forced expression of LNK.

Supplementary Figure 6

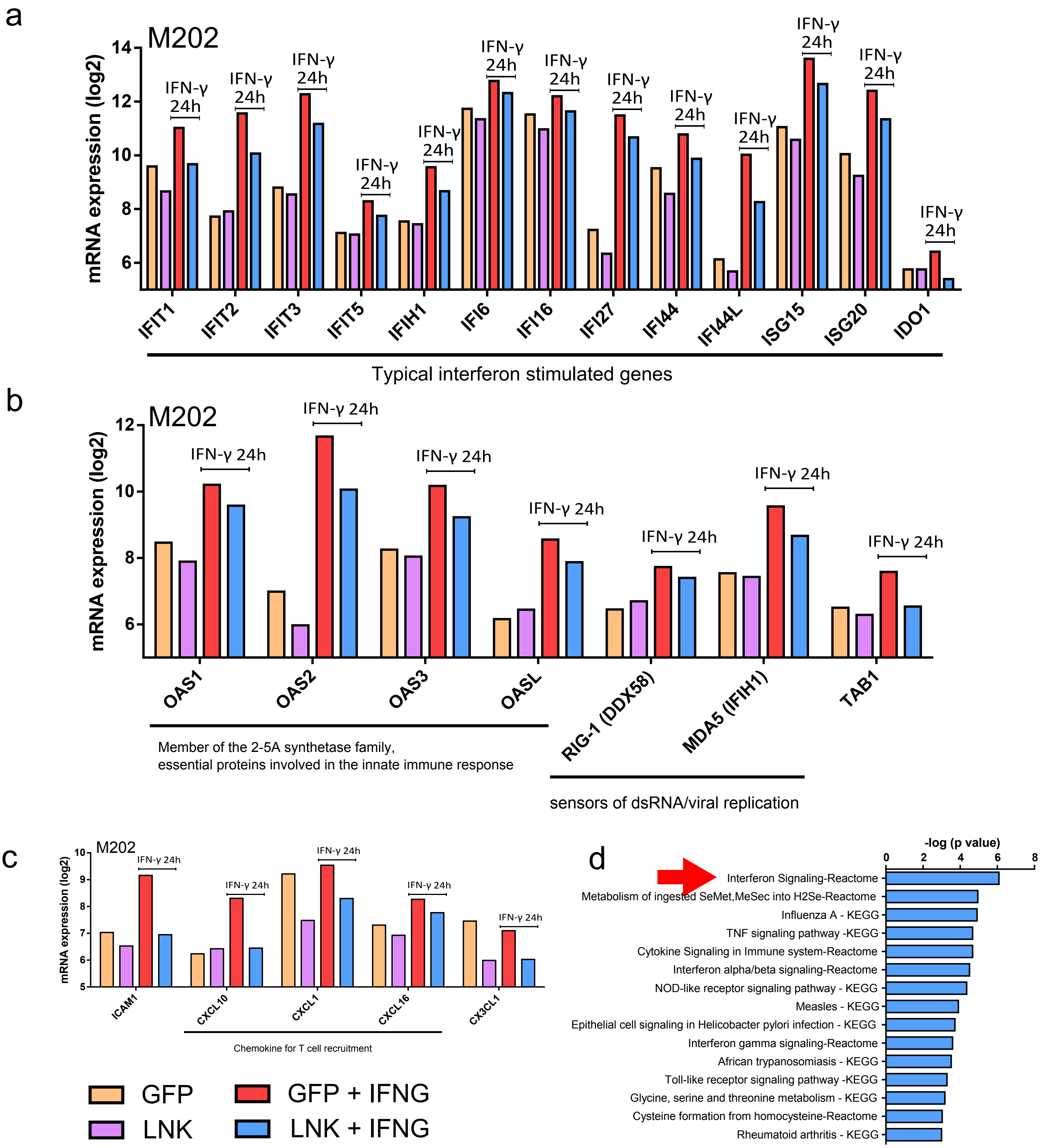

Supplementary Figure 6 LNK attenuates IFN-γ (2,000 U/ml, 24 hours) induced gene expression in M202 melanoma cells. **a** Force expression of LNK (LNK) suppresses the interferon simulated gene expression. Microarray analysis was performed to determine alterations of gene expression. **b** Force expression of LNK (LNK) suppresses expression of genes involved in innate immune response and dsRNA/viral sensors. **c** Force expression of LNK (LNK) suppresses expression of a number of chemokines involved in T cell recruitment. **d** Pathway analysis showed significantly enriched pathways in downregulating genes in M202 cells with force expressing of LNK and cultured with IFN gamma (2,000 U/ml, 24 hours).

Supplementary Figure 7

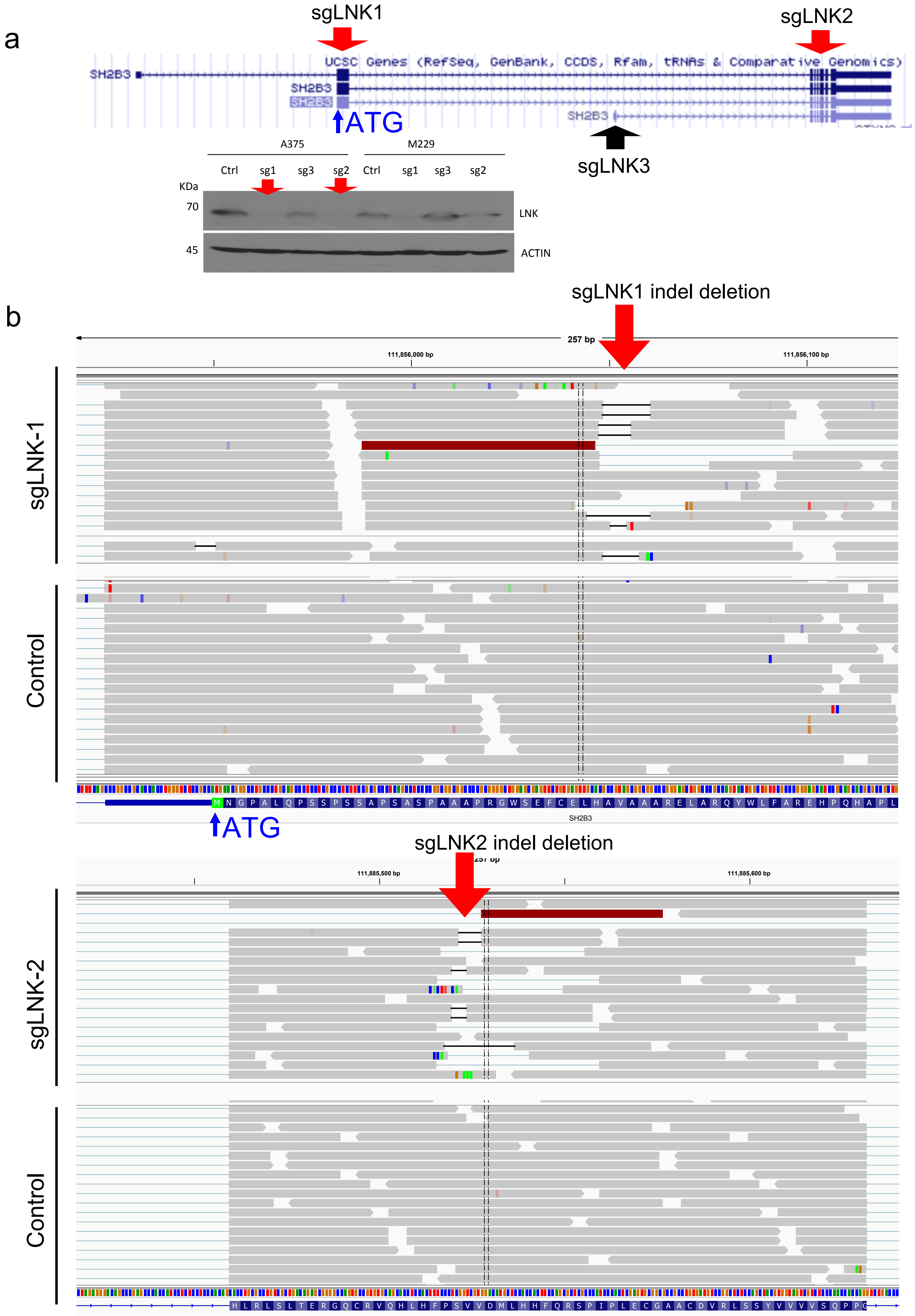

Supplementary Figure 7

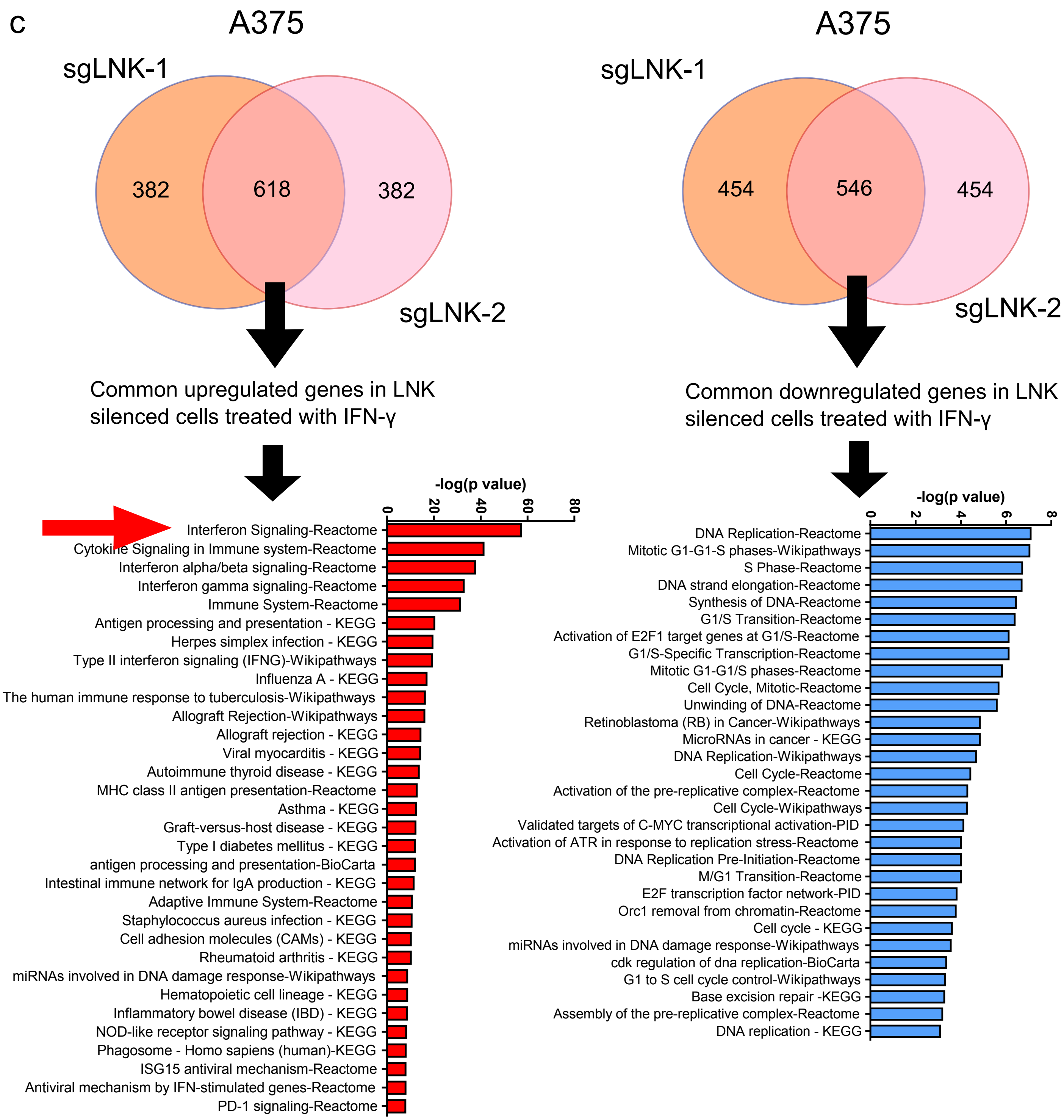

Supplementary Figure 7 Silencing LNK significantly enhanced interferon signaling in A375 melanoma cells. **a** Schematic diagram displays the CRISPR-Cas9 guide RNA targeting position. Knockdown efficiency was determined using western blot analysis. **b** Indel deletions induced by CRISPR-Cas9 using sgLNK-1 or sgLNK-2 were displayed using IGV program. **c** Pathway analysis of significantly enriched pathway in LNK silencing A375 cells using commonly either upregulated (left panel) or downregulated (right panel) genes in sgLNK-1 and sgLNK-2 cells.

Supplementary Figure 8

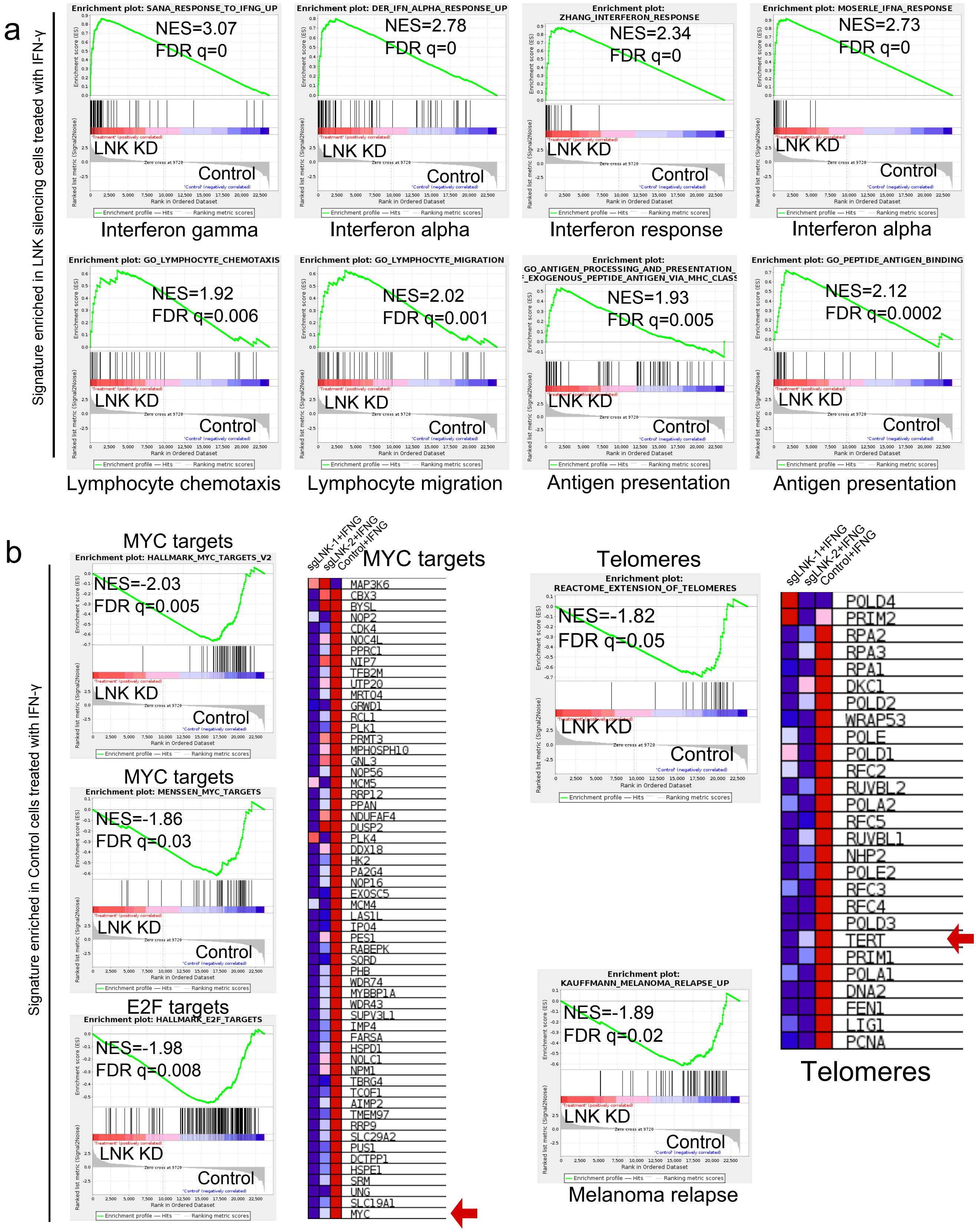

Supplementary Figure 8 Gene set enrichment analysis (GSEA) of the gene expression signature of LNK silenced A375 melanoma cells using CRISPR-Cas9. **a** Significant enriched gene expression signature in LNK silenced cells vs control cells. Both sets of cells were treated with IFN gamma for 24 hours (400 U/ml) (NES, normalized enrichment score; FDR, false discovery rate). The color scale indicates the positive (red) or negative (blue) correlation. **b** Significantly enriched gene expression signature in control cells vs LNK silenced cells. Both sets of cells were treated with IFN gamma for 24 hours (400 U/ml). The colors in heatmap indicates the expression values (red=high, pink=moderate, light blue=low, dark blue=lowest)

Supplementary Figure 9

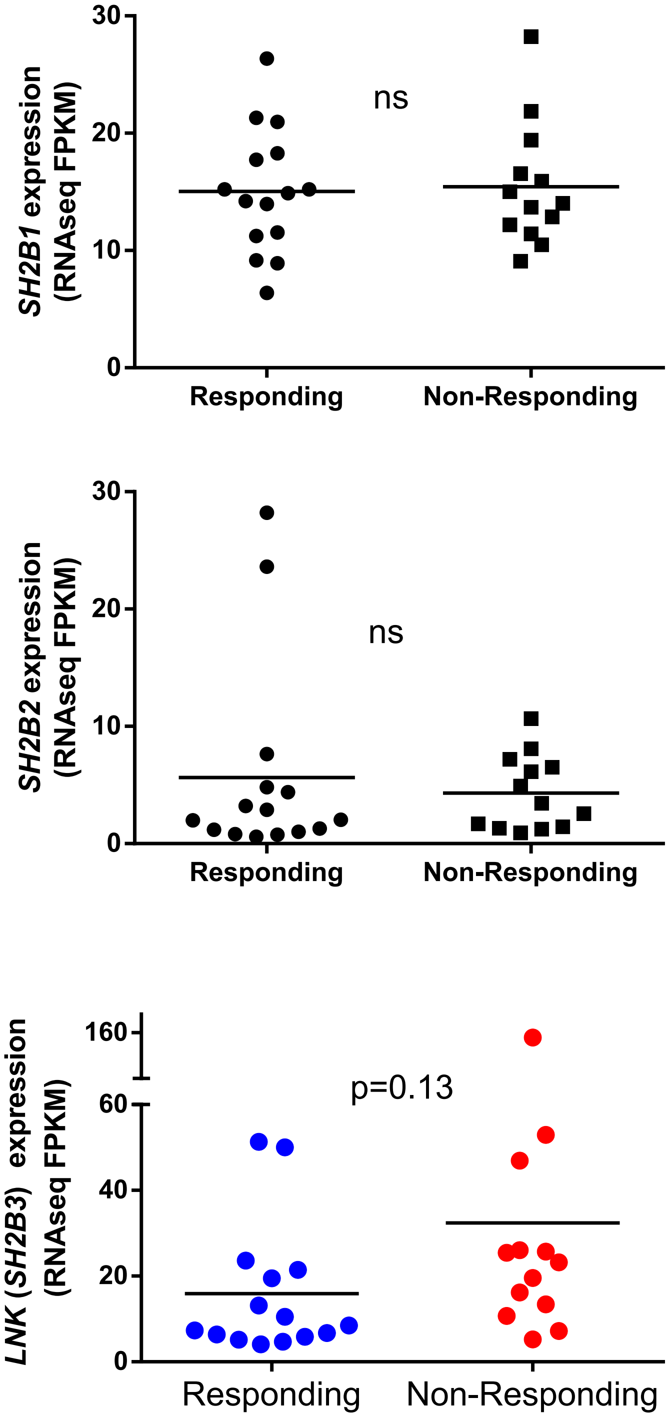

Supplementary Figure 9 mRNA expression levels of SH2B family members (*SH2B1*,*SH2B2* and *SH2B3*) in cohort of melanoma patients uniformly treated with PD-1 antibody (GSE78220). FPKM: Fragments Per Kilobase of transcript per Million mapped reads.

Supplementary Figure 10

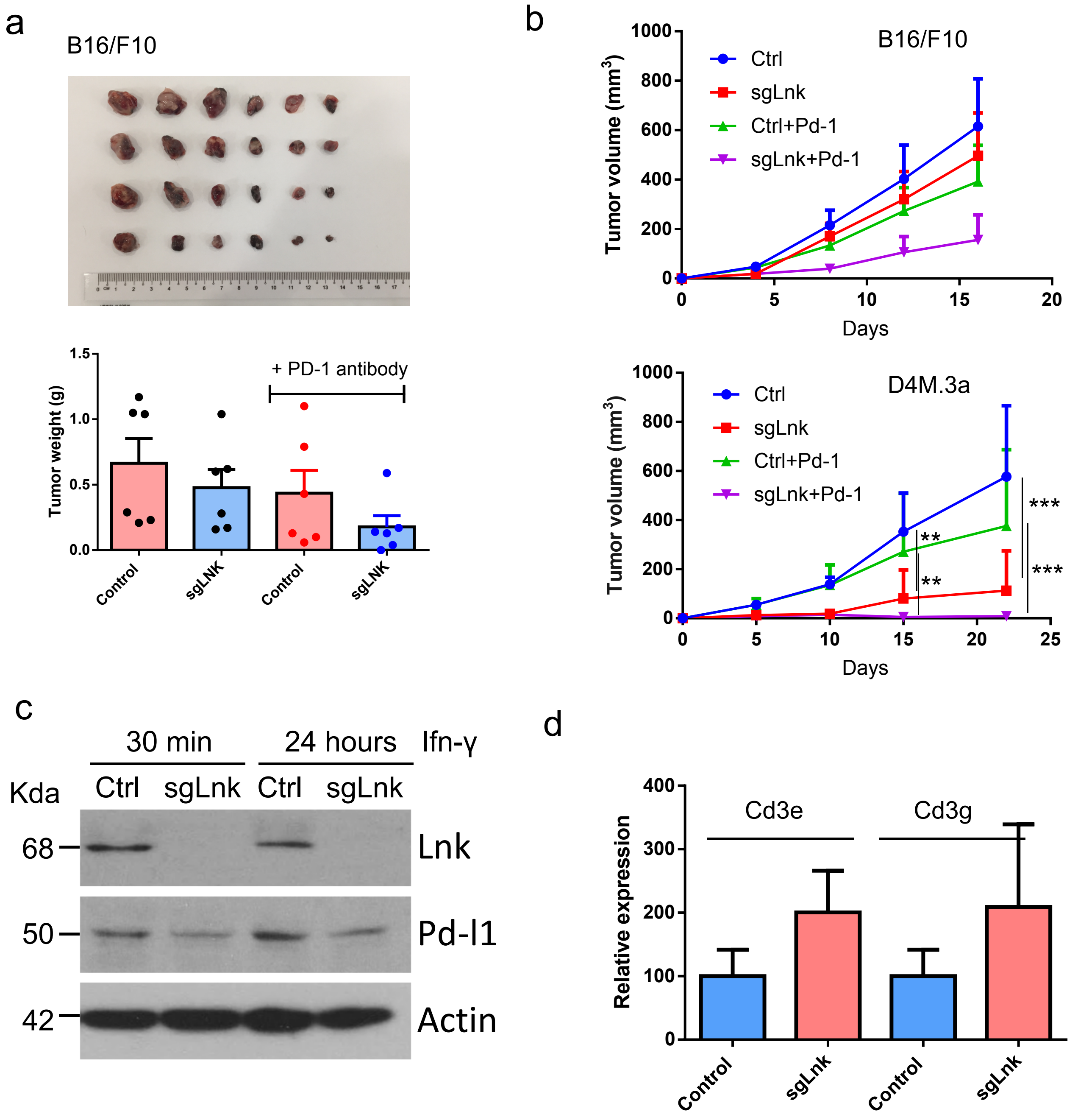

Supplementary Figure 10 Silencing Lnk in murine melanoma cells reduced tumor growth in immunocompetent mice. **a** Silencing Lnk in B16/F10 murine melanoma cells reduced tumor growth in immune competent C57BL mice. 0.05 million tumor cells were injected. **b** Tumor growth curves of B16/F10 and D4M.3A cells. **c** Western blot analysis of Pd-l1 expression in Lnk knockdown cells treated with murine Interferon gamma (400 U/ml, 30 min or 24 hours). **d** Real time PCR analysis of Cd3 expression in Lnk silenced tumors.

Supplementary Figure 11

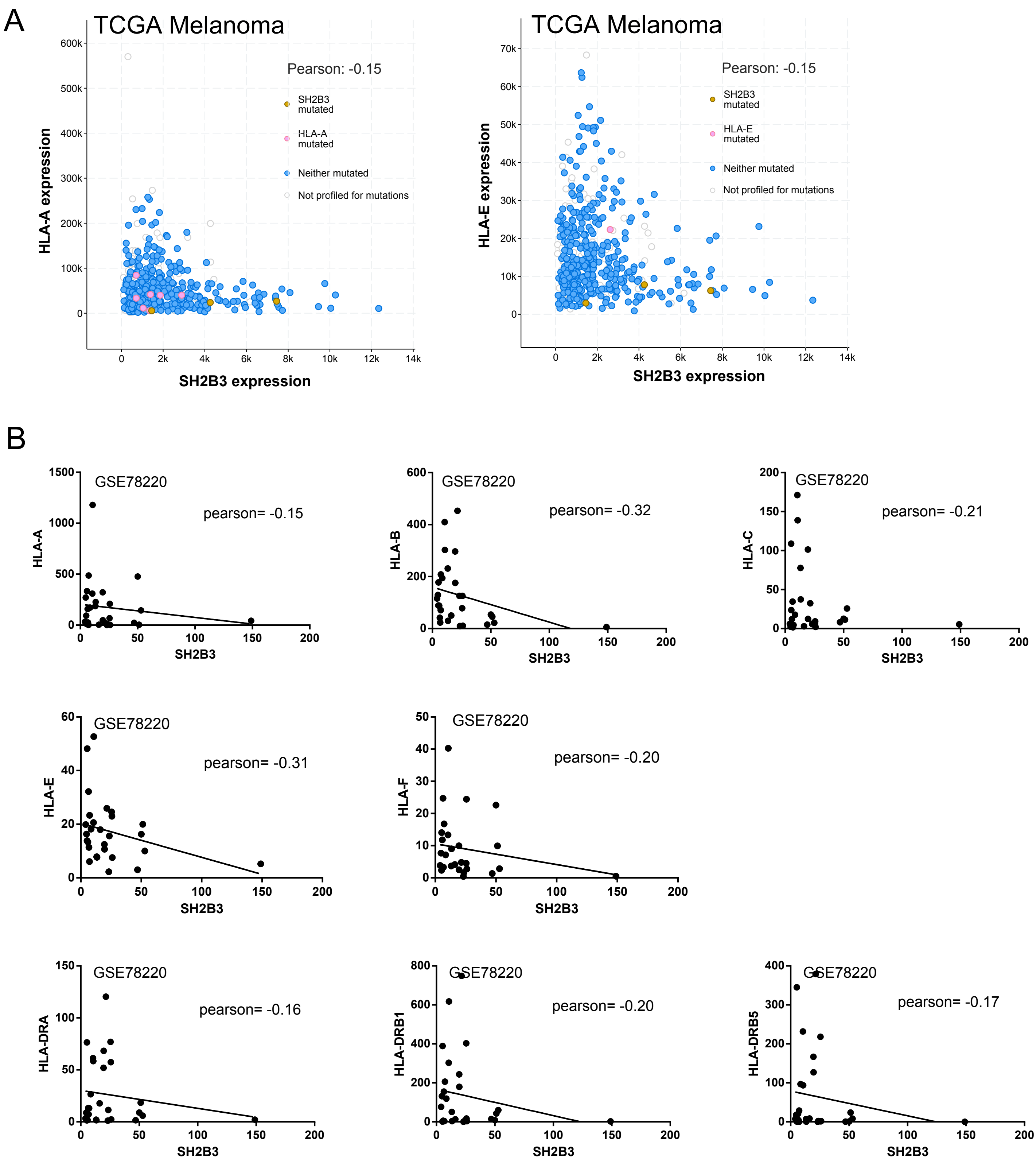

Supplementary Figure 11 *LNK* expression was negatively correlated with *HLA* genes expression in melanoma samples. **a** *LNK* expression levels were negatively correlated with expression level of *HLA-A* and *HLA-E* in TCGA melanoma samples. **b** *LNK* expression was negatively correlated with expression of HLA in melanoma samples (patient cohort GSE78220).

Supplementary Figure 12

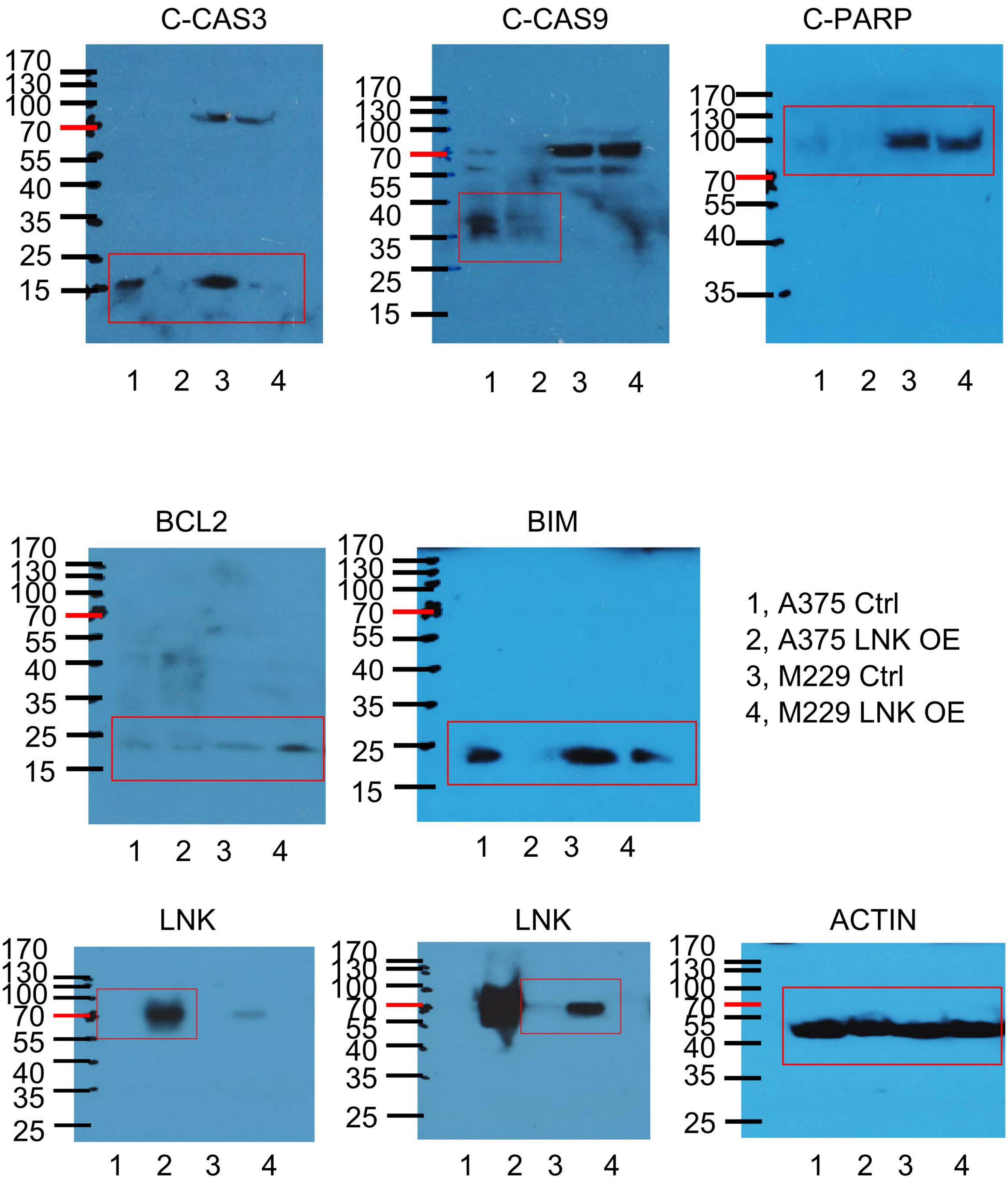

Figure 2g

Supplementary Figure 12

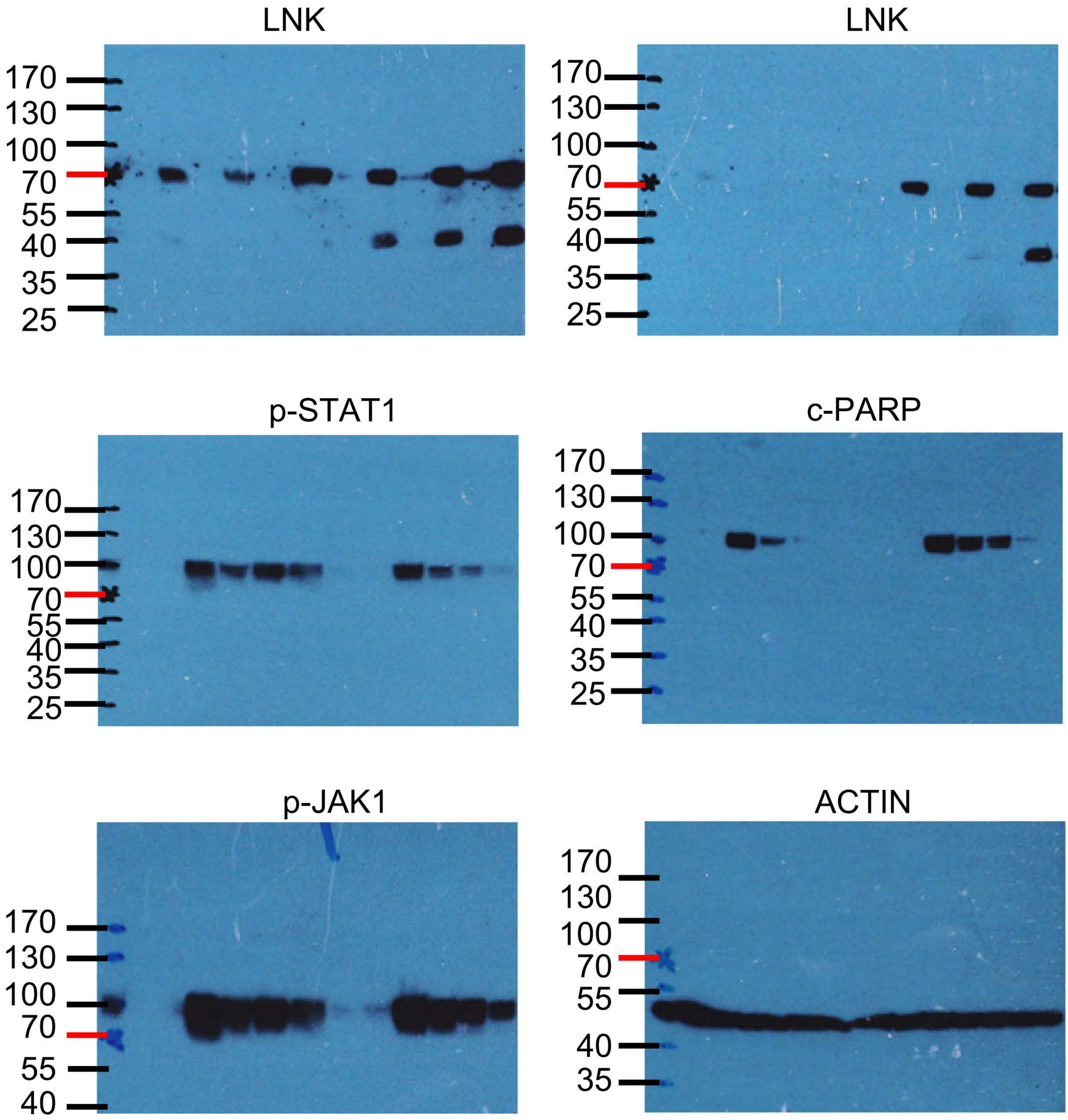

Figure 3a

Supplementary Figure 12

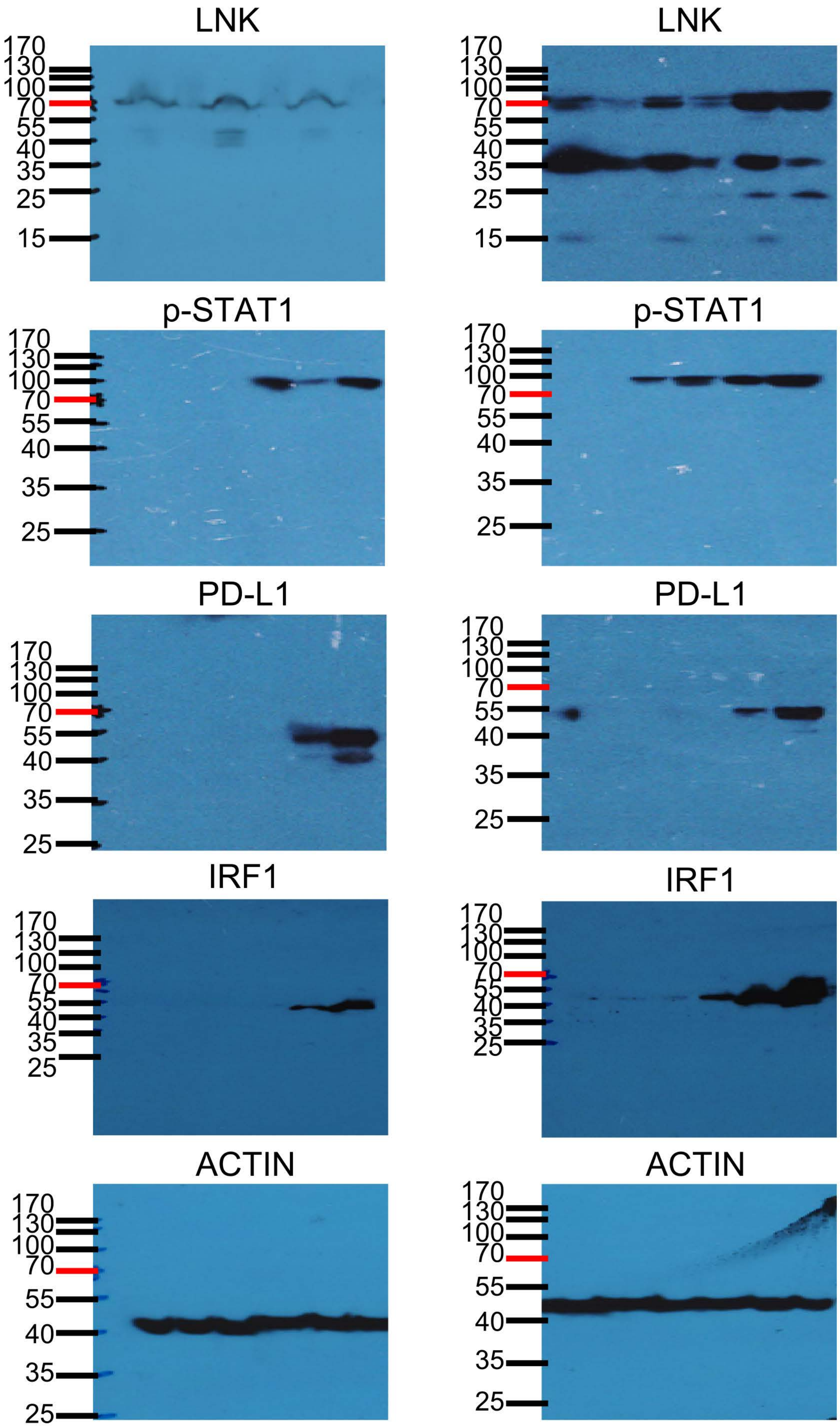

Figure 3b

Supplementary Figure 12

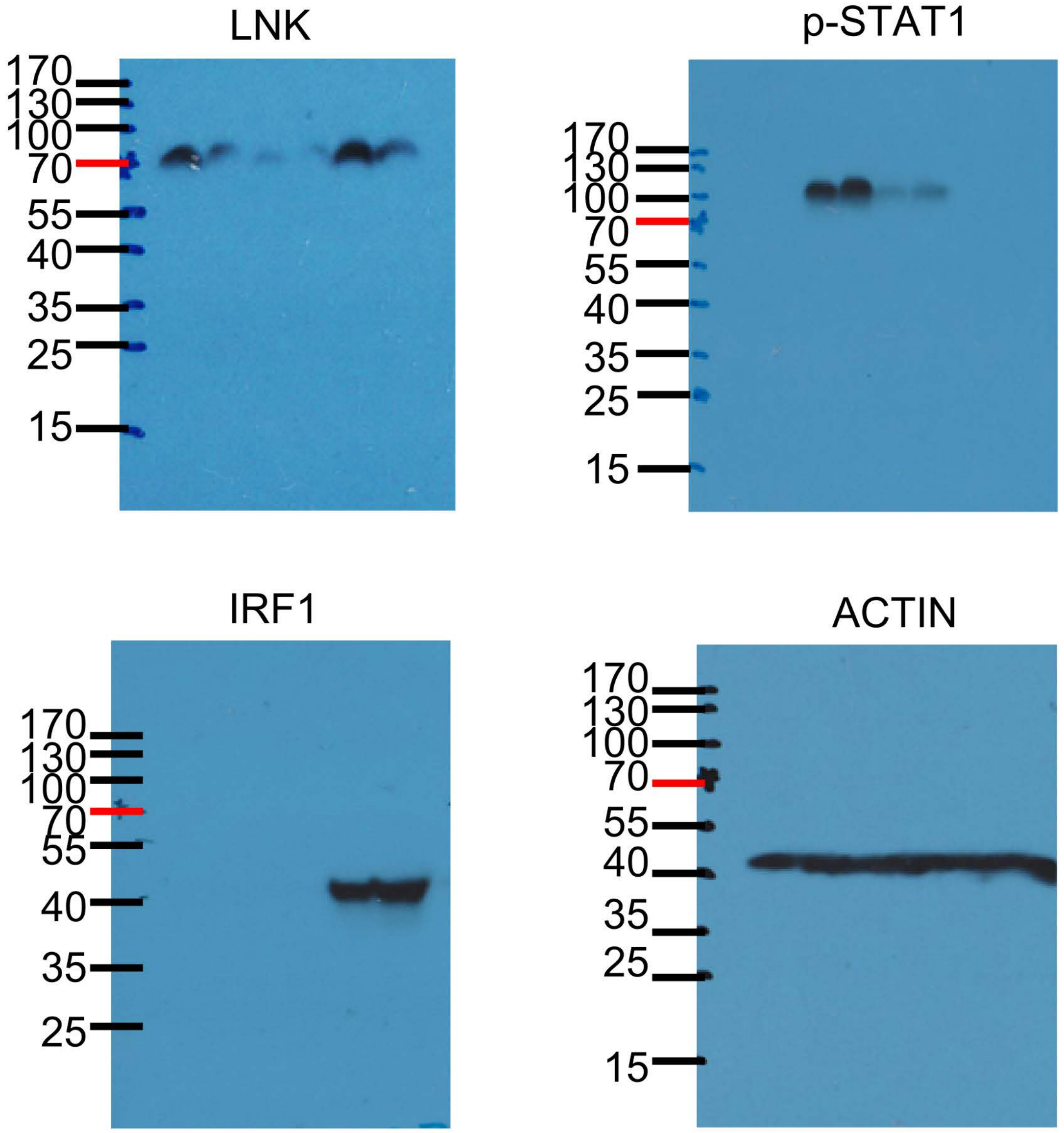

Figure 3c left panel

Supplementary Figure 12

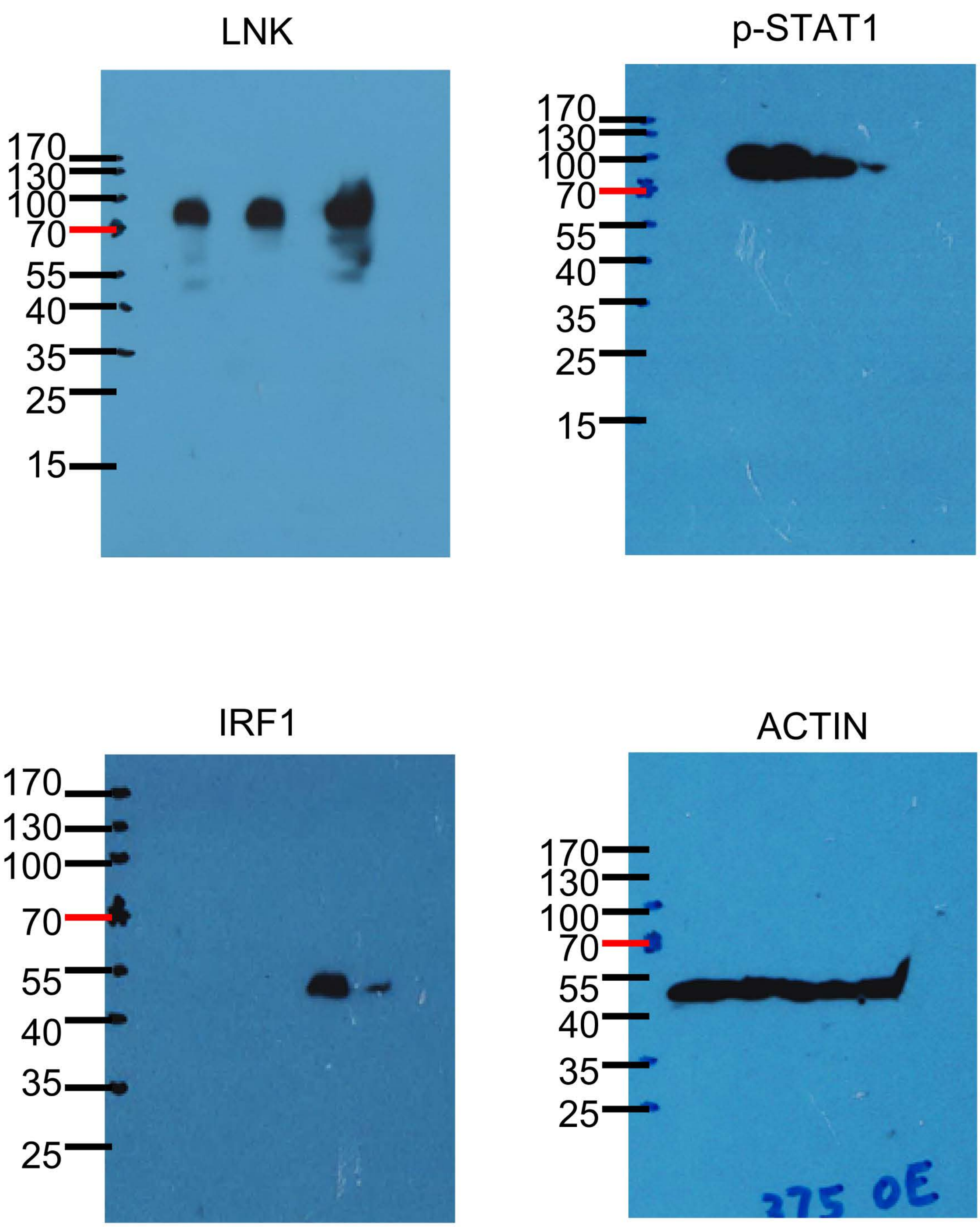

Figure 3c right panel

Supplementary Figure 12

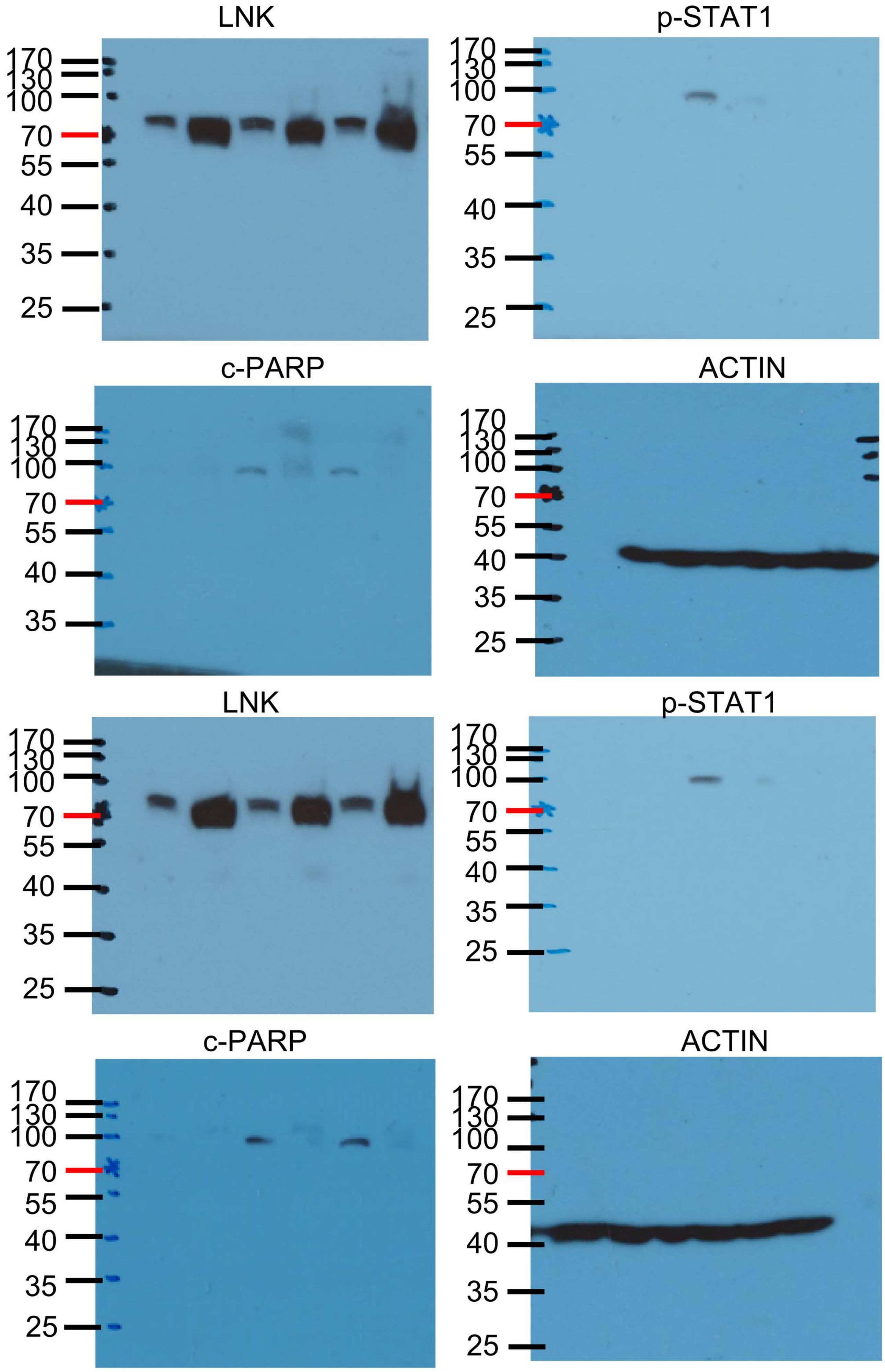

Figure 3d upper panel

Supplementary Figure 12

LNK

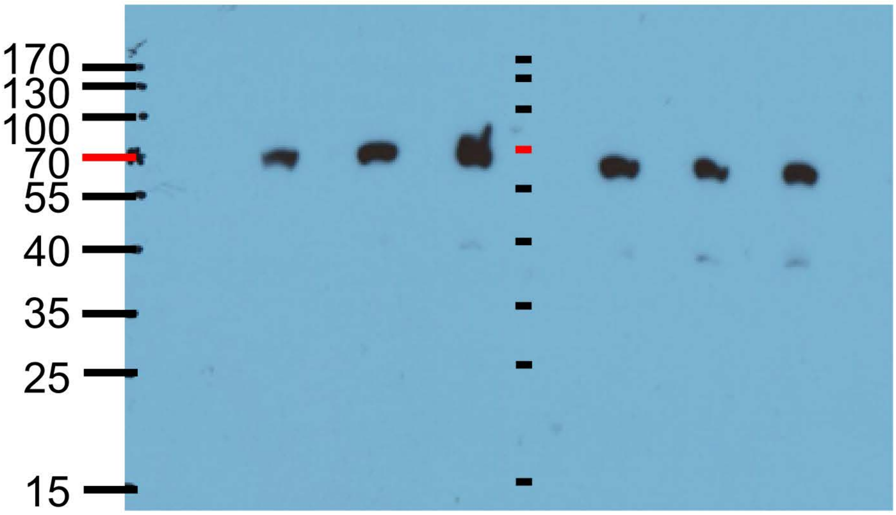

p-STAT1

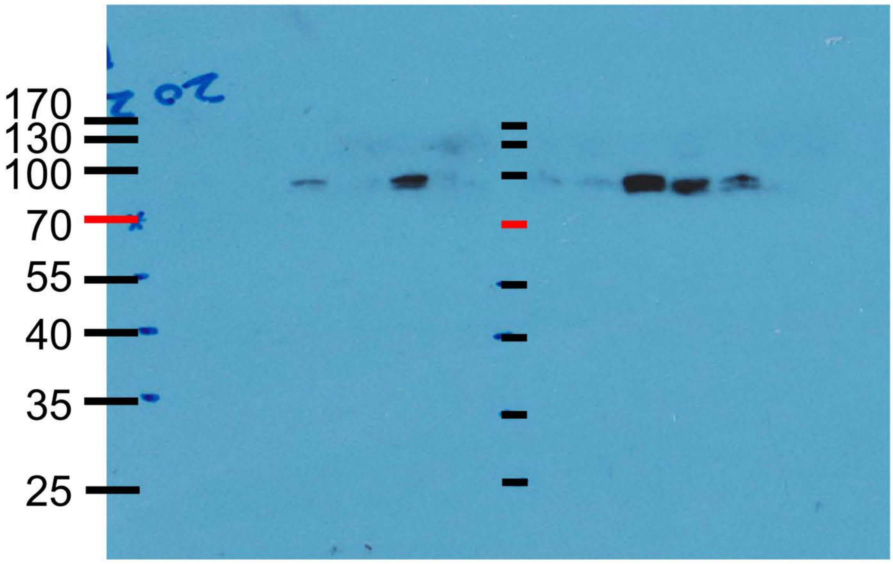

c-PARP

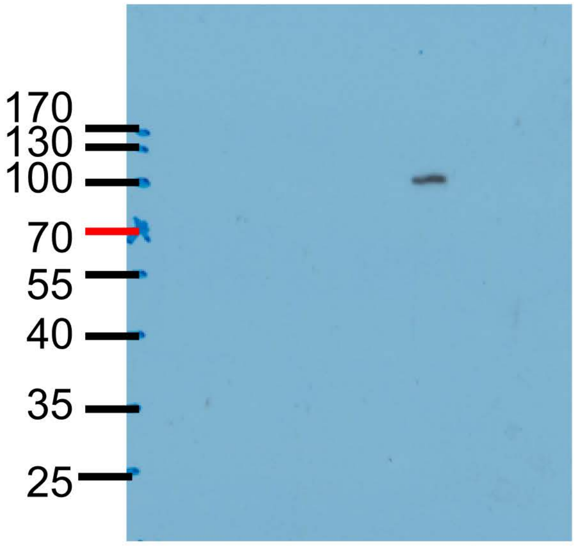

ACTIN

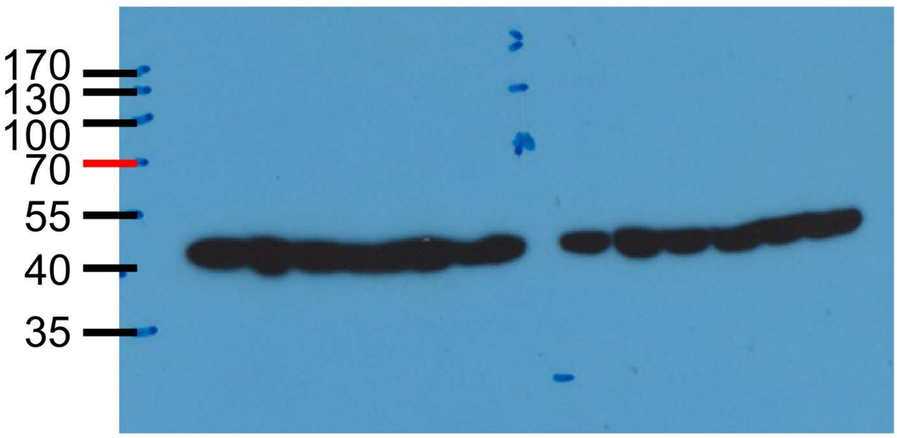

Figure 3d lower panel

Supplementary Figure 12

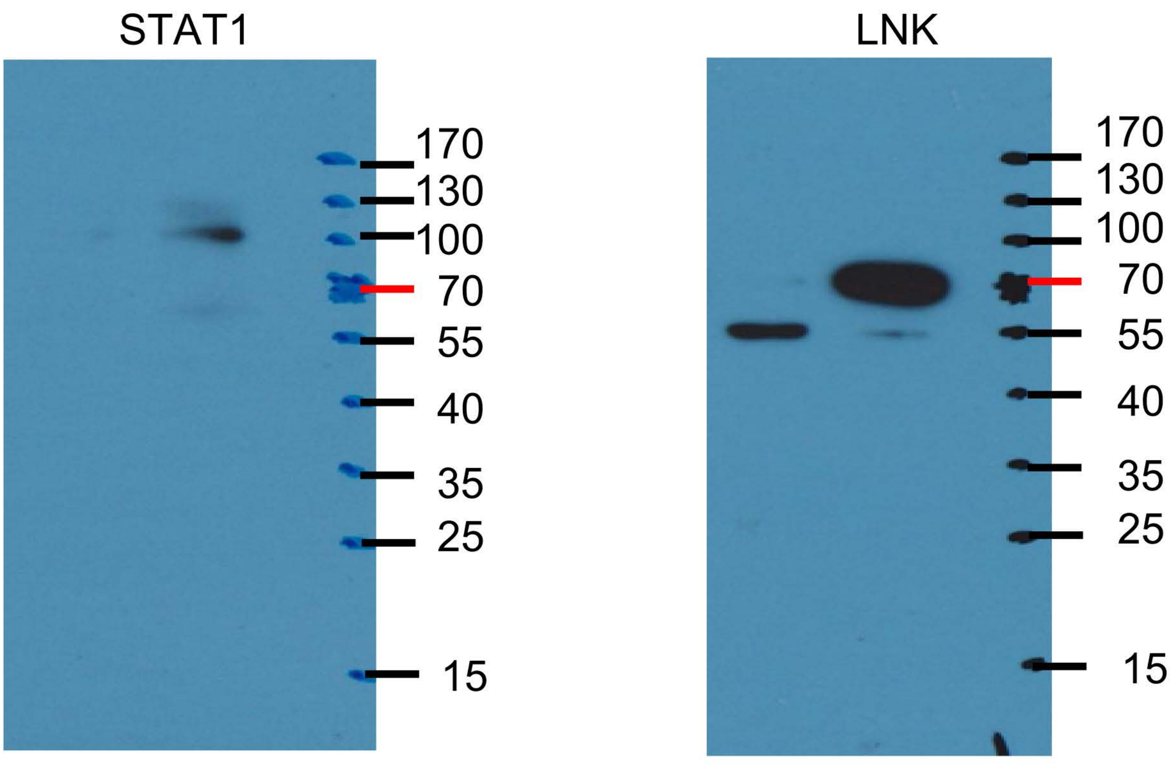

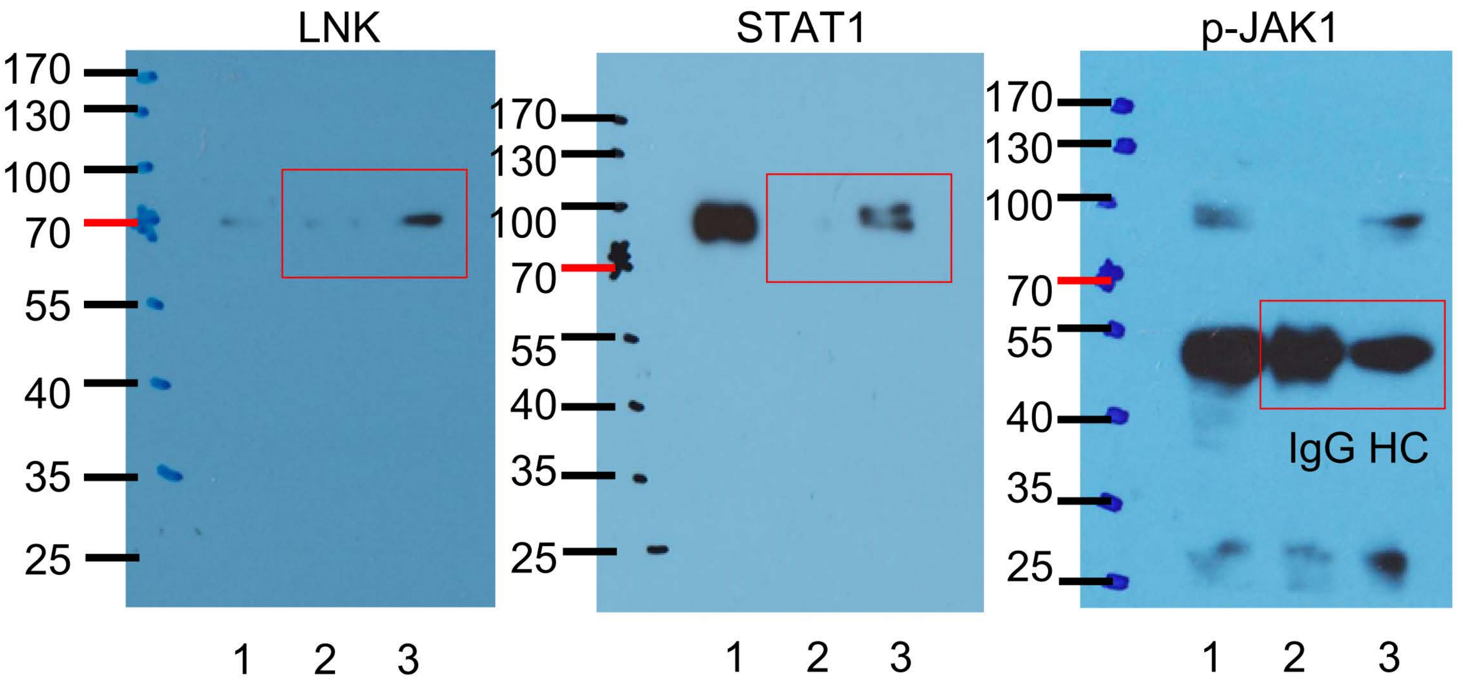

1, IP with PTPN2 antibody
2, IP with rabbit control IgG
3, IP with STAT1 antibody

Figure 3e

Supplementary Figure 12

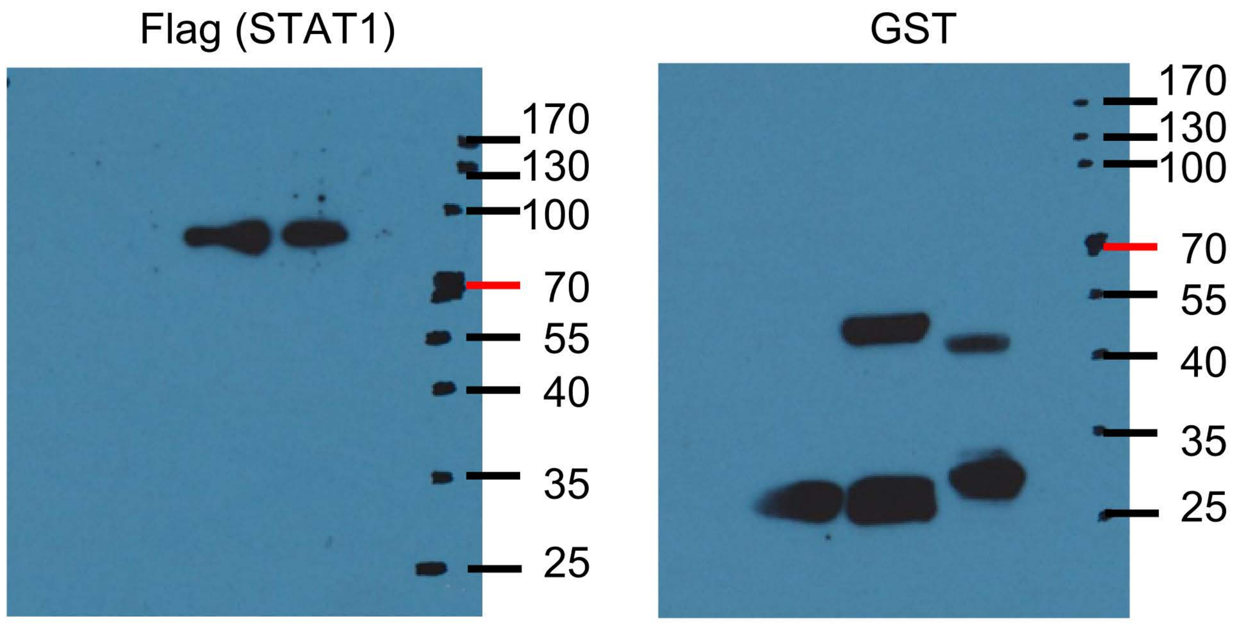

Figure 3f

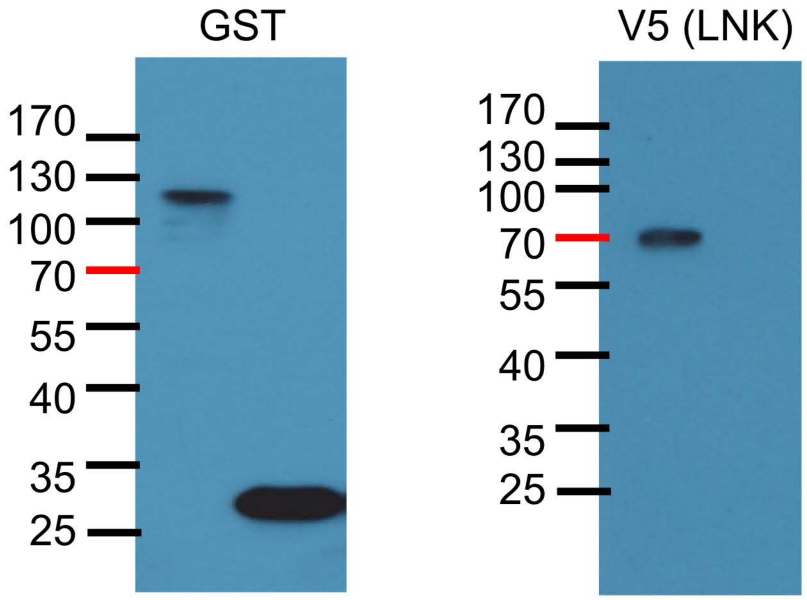

Figure 3g

Supplementary Figure 12

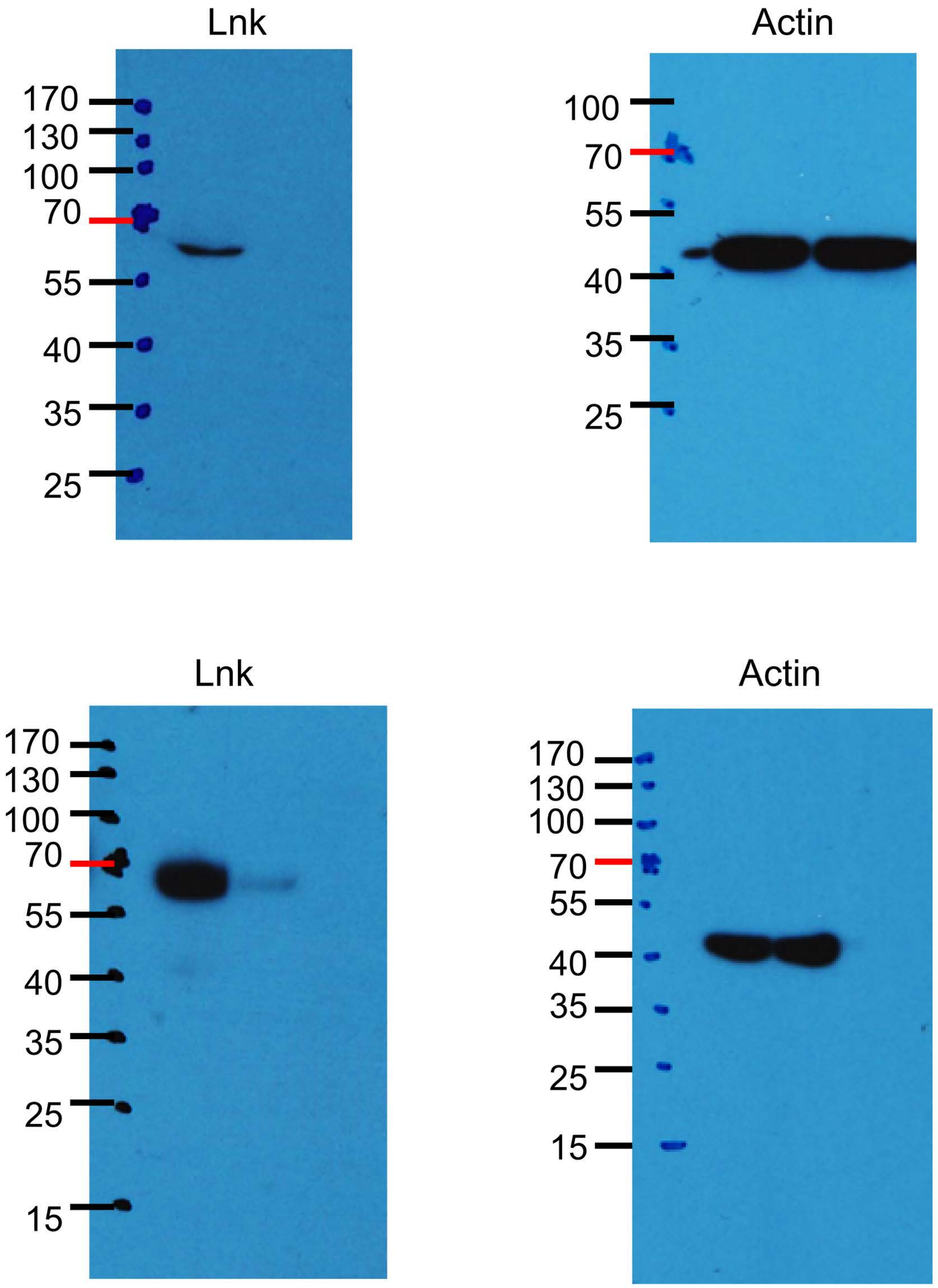

Figure 6f
