# Supplementary material for: LNK suppresses interferon signaling in melanoma
Source: Nat Commun. 2019 May 20;10:2230. doi: 10.1038/s41467-019-09711-y (PMC6527565; doi:10.1038/s41467-019-09711-y)
Supplement: Supplementary file 3 — Reporting Summary [file 41467_2019_9711_MOESM3_ESM.pdf]

nature research

Corresponding author(s): Qiao-Yang Sun

Last updated by author(s): Mar 14, 2019

Reporting Summary

Nature Research wishes to improve the reproducibility of the work that we publish. This form provides structure for consistency and transparency in reporting. For further information on Nature Research policies, see Authors & Referees and the Editorial Policy Checklist.

Statistics

For all statistical analyses, confirm that the following items are present in the figure legend, table legend, main text, or Methods section.

n/a	Confirmed	
☐	☒	The exact sample size (n) for each experimental group/condition, given as a discrete number and unit of measurement
☐	☒	A statement on whether measurements were taken from distinct samples or whether the same sample was measured repeatedly
☐	☒	The statistical test(s) used AND whether they are one- or two-sided<br>*Only common tests should be described solely by name; describe more complex techniques in the Methods section.*
☒	☐	A description of all covariates tested
☒	☐	A description of any assumptions or corrections, such as tests of normality and adjustment for multiple comparisons
☐	☒	A full description of the statistical parameters including central tendency (e.g. means) or other basic estimates (e.g. regression coefficient) AND variation (e.g. standard deviation) or associated estimates of uncertainty (e.g. confidence intervals)
☒	☐	For null hypothesis testing, the test statistic (e.g. F, t, r) with confidence intervals, effect sizes, degrees of freedom and P value noted<br>*Give P values as exact values whenever suitable.*
☒	☐	For Bayesian analysis, information on the choice of priors and Markov chain Monte Carlo settings
☒	☐	For hierarchical and complex designs, identification of the appropriate level for tests and full reporting of outcomes
☒	☐	Estimates of effect sizes (e.g. Cohen's d, Pearson's r), indicating how they were calculated

Our web collection on statistics for biologists contains articles on many of the points above.

Software and code

Policy information about availability of computer code

Data collection	N.A.
Data analysis	GraphPad Prism 6 was used for the statistical analysis. RNA-seq aligned to hg19 genome and UCSC reference transcript using STAR (version 2.4.0). Expression analysis performed by Featurecounts (version 1.5.0). Bigwig was generated after normalization. For microarry, the data were quantile normalized followed by log2 transformed using beadarray Bioconductor package.

For manuscripts utilizing custom algorithms or software that are central to the research but not yet described in published literature, software must be made available to editors/reviewers. We strongly encourage code deposition in a community repository (e.g. GitHub). See the Nature Research guidelines for submitting code & software for further information.

Data

Policy information about availability of data

All manuscripts must include a data availability statement. This statement should provide the following information, where applicable:

- Accession codes, unique identifiers, or web links for publicly available datasets
- A list of figures that have associated raw data
- A description of any restrictions on data availability

The RNA sequencing and microarray data has been deposited in the GEO database under the accession code GSE127764, GSE127333 and GSE127965. All the other data supporting the findings of this study are available within the article and its supplementary information files and from the corresponding author upon reasonable request. A reporting summary for this article is available as a Supplementary Information file.

Field-specific reporting

Please select the one below that is the best fit for your research. If you are not sure, read the appropriate sections before making your selection.

☒ Life sciences ☐ Behavioural & social sciences ☐ Ecological, evolutionary & environmental sciences

For a reference copy of the document with all sections, see nature.com/documents/nr-reporting-summary-flat.pdf

Life sciences study design

All studies must disclose on these points even when the disclosure is negative.

Sample size	For the murine xenograft experiment, a sample size of n ≥ 6 per group was used to achieve a statistical significance of $p<0.05$.
Data exclusions	No data were excluded from analysis. For murine experiment, all tumor samples were included in the analysis.
Replication	Data are representatives of three independent experiments with similar results.
Randomization	For the murine xenograft Pd1 treatment experiment, Mice were randomly separated into two group (either Pd-1 treated or untreated group)
Blinding	Experiments were performed in a non-blinded way.

Reporting for specific materials, systems and methods

We require information from authors about some types of materials, experimental systems and methods used in many studies. Here, indicate whether each material, system or method listed is relevant to your study. If you are not sure if a list item applies to your research, read the appropriate section before selecting a response.

Materials & experimental systems

n/a	Involved in the study	
☐	☒	Antibodies
☐	☒	Eukaryotic cell lines
☒	☐	Palaeontology
☐	☒	Animals and other organisms
☒	☐	Human research participants
☒	☐	Clinical data

Methods

n/a	Involved in the study	
☒	☐	ChIP-seq
☒	☐	Flow cytometry
☒	☐	MRI-based neuroimaging

Antibodies

Antibodies used	anti-human LNK antibody (AF5888, R&D system Inc, 1:2000); anti-mouse/human Lnk/LNK ((A-12) sc-393709, Santa Cruz Biotechnology Inc, 1:500); anti-β-actin (a1978, Sigma-Aldrich, 1:6000); anti-p-JAK2 (Tyr1007/1008, 3776S, Cell Signaling Technology Inc, 1:2500), JAK2 (3230S, Cell Signaling Technology Inc, 1:2500), p-JAK1 (Tyr1022/1023, 3331, Cell Signaling Technology Inc, 1:2500), JAK1 (3344, Cell Signaling Technology Inc, 1:2500), p-STAT1 (Tyr701, 7649, Cell Signaling Technology Inc, 1:2500), STAT1 (9172, Cell Signaling Technology Inc, 1:2500), IRF1 (8478, Cell Signaling Technology Inc, 1:2500), Cleaved Caspase-3 (9661, Cell Signaling Technology Inc, 1:2000), Cleaved Caspase-9 (9501, Cell Signaling Technology Inc, 1:2000), BCL2 (4223, Cell Signaling Technology Inc, 1:2000), BIM (2933, Cell Signaling Technology Inc, 1:2000), c-PARP (Asp214, 5625, Cell Signaling Technology Inc, 1:2500), PD-L1 (13684, Cell Signaling Technology Inc, 1:2000) and GAPDH (2118, Cell Signaling Technology Inc, 1:6000).
Validation	Antibodies were selected using validated antibodies portal antibodypedia (https://www.antibodypedia.com/). LNK antibody were validated with shRNA and CRISPR knockdown experiments and examined by western blot analysis.

Eukaryotic cell lines

Policy information about cell lines

Cell line source(s)	Melanoma cell line A375 and B16/F10 were from ATCC; and primary human melanoma cells (M202, M229, M238, M285, M368) were established and kindly provided by Dr Antoni Ribas, UCLA. Murine melanoma cell line D4M.3A was established and provided by Dr David W.Mullins.
Authentication	Cells were obtained from ATCC and stored in liquid nitrogen and low passage cells were used for the experiment. Primary human melanoma cells were obtained from Dr Antoni Ribas who established the cell lines. Murine melanoma cell line D4M.3A was established and provided by Dr David W.Mullins.

Mycoplasma contamination	Mycoplasma was tested using PCR.
Commonly misidentified lines (See ICLAC register)	Non misidentified cell lines used in this study.

Animals and other organisms

Policy information about studies involving animals; ARRIVE guidelines recommended for reporting animal research

Laboratory animals	For human melanoma cell lines, 5-8 week old male Nod-SCID mice were used for the xenograft experiments. For murine melanoma cell line B16/F10 and D4M.3A, 8-12 week old syngeneic male C57BL/6 mice were used for the experiments.
Wild animals	No wild animals were used in this study.
Field-collected samples	No field collected samples were used in this study.
Ethics oversight	The animal study was conducted according to the ethical regulations and was approved (R18-0450) by NUS Institutional Animal Care and Use Committee (IACUC).

Note that full information on the approval of the study protocol must also be provided in the manuscript.

October 2018
